# Prescription of antibiotics to children with acute otitis media in Danish general practice

**DOI:** 10.1186/s12875-020-01248-0

**Published:** 2020-08-27

**Authors:** Jonas K. Olsen, Jesper Lykkegaard, Malene Plejdrup Hansen, Frans B. Waldorff, Jørgen Lous, Merethe K. Andersen

**Affiliations:** 1 Research Unit for General Practice, Institute of Public Health, University of Southern Denmark, JB Winsløws Vej 9A, DK-5000 Odense C, Denmark; 2 Audit Project Odense, Research Unit for General Practice, Institute of Public Health, University of Southern Denmark, Odense, Denmark; 3 Center for General Practice at Aalborg University, Aalborg, Denmark

**Keywords:** Antibacterial agents, Child, General practice, Guideline adherence, Otitis media

## Abstract

**Background:**

Acute otitis media (AOM) is a common and most often self-limiting infection in childhood, usually managed in general practice. Even though antibiotics are only recommended when certain diagnostic and clinical criteria are met a high antibiotic prescription rate is observed.

The study’s objective was to analyse associations between patient- and general practitioner (GP) characteristics and antibiotic prescribing for children with AOM in an effort to explain the high antibiotic prescribing rates.

**Methods:**

All general practices in the Northern, Southern and Central regions of Denmark were invited to record symptoms, examinations, findings and antibiotic treatment for all children ≤7 years of age diagnosed with AOM during a four-week winter period in 2017/2018. Associations were analysed by means of multivariate logistic regressions. The study design was cross-sectional.

**Results:**

GPs from 60 general practices diagnosed 278 children with AOM of whom 207 (74%) were prescribed antibiotics, most often penicillin V (60%). About half of the children had tympanometry performed. Antibiotic prescribing rates varied considerably between practices (0–100%). Antibiotic prescribing was associated with fever (odds ratio (OR) 3.69 95% confidence interval (CI) 1.93–7.05), purulent ear secretion (OR 2.35 95% CI 1.01–5.50) and poor general condition (OR 3.12 95% CI 1.31–7.46), and the practice’s antibiotic prescribing rate to other patients with symptoms of an acute respiratory tract infection (OR 2.85 CI 95% 1.07–7.60) and specifically to other children with AOM (OR 4.15 CI 95% 1.82–9.47).

**Conclusion:**

GPs’ antibiotic prescribing rates for children with AOM vary considerably even considering the of signs, symptoms, request for antibiotics, and use of tympanometry. Interventions to reduce overprescribing should be targeted high-prescribing practices.

## Background

Acute otitis media (AOM) is a common and most often self-limiting infection in childhood, usually managed in general practice. In Denmark, 60% of all children will have experienced at least one episode of AOM before their seventh birthday [1]. AOM diagnostics is based on a history of acute otalgia, fever, malaise, irritability, and sleep problems in combination with characteristic otoscopy and tympanometry findings with sign of fluid in the middle ear [2]. Immediate antibiotics are recommended for infants younger than 6 months, children younger than 2 years with bilateral infection, children with purulent secretion from the ear, and children with poor general condition and severe symptoms [2–4]. For all other children guidelines recommend analgesic treatment and watchful waiting for up to 3 days [2–4].

In cases of AOM, antibiotics more often result in adverse events than analgesics, such as vomiting, diarrhoea or rash [5, 6]. Furthermore, bacterial resistance to antibiotics is an increasing problem and every antibiotic prescription potentially worsens this development [7]. Pathogens causing AOM are responsible for other illnesses where the existence of effective antibiotics is crucial (e.g. pneumonia) [8]. O’Neill and colleagues predicts deaths due to infections with antibiotic resistant bacteria to reach ten million lives each year world-wide by year 2050 [9]. In European countries, primary care is responsible for about 90% of all antibiotic prescriptions [8, 10]. It has been estimated that if the recommendations were followed less than 50% of children with AOM in general practice should be prescribed antibiotics [11]. However, prescription rates are much higher (84%) [12] warranting interventions to reduce overuse of antibiotics. Such interventions could be more properly targeted if it was known whether overprescribing of antibiotics for children with AOM is a universal problem in general practice or mainly restricted to high prescribers [13].

This study aimed to analyse associations between patient- and general practitioner (GP) characteristics and antibiotic prescribing for children with AOM in an effort to explain the high antibiotic prescribing rates.

## Methods

### Design

This study is based on cross-sectional data from two audits on management of patients with acute respiratory tract infections (RTI) conducted in the winters of year 2017 in the Northern and Southern region and year 2018 in the Central region of Denmark. All GP practices in the regions were invited. By use of the Audit Project Odense (APO) method, for 20 days, the participating GPs and staff consecutively registered all patient encounters with RTI including AOM. The recording method is well-known by Danish GPs and has been used in several other studies [14]. Only first consultations with an episode of RTIs were included and patients currently treated with antibiotics were excluded. According to other studies on AOM only children aged ≤7 years was included [1, 15].

### Setting

Virtually all services in the Danish healthcare system are fully tax-funded. GPs act as primary care providers and gatekeepers to more extended services (e.g. at hospitals) [16]. As an exception consulting an oto-rhino-laryngologists does not require a GP referral. Nevertheless, the GPs manage most cases of AOM. Danish GPs are paid capitation fees (1/3) and fees for services (2/3) [16], including a fee for performing a tympanometry (~ 14 EUR) [17]. Denmark has one of the lowest antibiotic prescribing rates in Europe [18]. In Danish general practice, patients with RTIs are often managed by GP trainee doctors and practice nurses working under the supervision of the principal GP(s) [19].

### Data

For each patient a questionnaire was filled in (supplementary figure 1) by the healthcare professional. Among the recorded items were age, gender, duration of symptoms, symptoms (worsening following temporary improvement, fever, purulent otorrhea and ear/face pain), clinical findings (poor general condition), examination results (abnormal or normal tympanometry), diagnosis, antibiotic treatment (penicillin V, amoxicillin +/÷ clavulanic acid, macrolide, other antibiotic and no antibiotic) and other (patient requesting antibiotics). Age and days with symptoms were counted in whole numbers and all other variables were ticked off if considered present by the healthcare professional. The healthcare professionals received no additional education regarding diagnostic criteria or treatment before enrolling patients in the study. Each participating practice reported on the age and gender of the GPs working in the practice and the practice’s location in Denmark.

### Analyses

A multivariate logistic regression was used to analyse associations between patient characteristics and being diagnosed with AOM. The primary analyses were four multi-adjusted models developed to analyse factors associated with antibiotic prescribing in children with AOM: 1) included the patient’s age, gender, symptoms and signs. 2) included model 1 and added the GP’s age, gender and region of practice. In model 3 and 4 each child was further characterized by the general practice’s antibiotic prescribing rates, respectively, for patients with RTI in general and specifically for children with AOM. These were individually respectively computed as the proportion of the other recorded patients with RTI and children with AOM in the practice who had antibiotics prescribed. The rates were divided in lowest 25th percentile, 25th–75th percentile and highest 75th percentile. The practice’s rate of antibiotic prescription for children with AOM could not be assessed for 15 children in practices, as these practices only registered one child with AOM. These practices and children were excluded from the fourth model. The models were based on diagnostic criteria of AOM [2] and results from univariate analysis.

In the analyses, it was assumed that GP trainee doctors and practice nurses acted in accordance with and under the supervision of the principal GP(s). Consequently, children managed by a nurse or GP trainee were classified according to the principal GP(s)‘s gender and age.

Standard errors in the models are specified as robust. A test for variance inflation factor (VIF) was performed to ensure noncollinearity with standard threshold of VIF ≤ 10. Missing values were removed listwise. All analyses were performed using STATA 15.1 statistical software (StataCorp, Texas, USA).

## Results

All 1899 GPs in the Northern (*N* = 303), Southern (*N* = 785) and Central (*N* = 811) regions of Denmark were invited to participate in the study. In total 143 (7.5%) GPs participated, of whom 85 (59%) diagnosed at least one child with AOM. The number of children registered with AOM varied between GPs from 0 to 6 for the 5th and 95th percentile, respectively. The average age of the participating GPs was lower than the average in the Northern region, but equal to the regional average in the Southern and Central regions. The proportion of female GPs was higher compared to the background population of GPs in the Southern region [20–22] (Table 1).

**Table 1:** Characteristics of the 143 participating GPs compared to all GPs in the three regions (2017–2018)

	Northern Region n (%) [95% CI]		Southern Region n (%) [95% CI]		Central Region n (%) [95% CI]	
	*All GPs*^a^	Participant GPs	All GPs^a^	Participant GPs	All GPs^a^	Participant GPs
	303	59 (19.5)	785	46 (5.9)	811	38 (4.7)
Age (mean)	53.9	49.0 [46.4–51.6]	59.1	49.1 [46.3–51.9]	51.7	50.7 [47.8–53.7]
< 50 years	108 (35.6)	35 (59.3) [45.7–71.9]	353 (45.0)	24 (52.2) [36.9–67.1]	359 (44.3)	20 (52.6) [35.8–69.0]
≥ 50 years	195 (64.4)	24 (40.7) [28.1–54.3]	432 (55.0)	22 (47.8) [32.9–63.1]	452 (55.7)	18 (47.4) [31.0–64.2]
Sex						
Male	169 (55.8)	24 (40.7) [28.1–54.3]	400 (51.0)	16 (34.8) [21.4–50.2]	384 (47.3)	14 (36.8) [21.8–54.0]
Female	134 (44.2)	35 (59.3) [45.7–71.9]	385 (49.0)	30 (65.2) [49.8–78.6]	427 (52.7)	24 (63.2) [46.0–78.2]

*GP* General practitioner, *CI* Confidence interval

^a^numbers from the Association of Danish GPs (PLO)’ fact sheet

The study included 278 children diagnosed with AOM without missing data (47 cases excluded due to missing data). Compared to children diagnosed with other RTIs, the AOM diagnosis was associated with purulent ear secretion, ear pain, abnormal tympanometry, and fever (Supplementary Table 1).

Slightly more boys than girls were diagnosed with AOM (56%). Mean age was 2.5 years. Tympanometry was performed in about half of the cases (Table 2).

**Table 2:** Characteristics of children aged 0–7 years diagnosed with AOM in Danish general practice (2017–2018)

Variables	AOM ***n*** (%) [95% CI])
N	278 (100.0)
**Gender**	
Boys	157 (56) [50.4–62.4]
Girls	121 (44) [37.6–49.6]
**Age**	
Mean	2.5 [2.3–2.8]
< 2 years	121 (44) [37.6–49.6]
≥ 2 years	157 (56) [50.4–62.4]
Symptom duration ≤3 days	133 (48) [41.8–53.9]
**Symptoms**	
Symptom worsening	42 (15) [11.1–19.9]
Fever (temp. > 38.5 °)	175 (63) [57.0–68.6]
Purulent ear secretion	58 (21) [16.2–26.1]
Ear/face pain	178 (64) [58.1–69.7]
**Clinical Findings**	
Poor general condition	62 (22) [17.5–27.7]
**Tympanometry**	144 (52) [45.8–57.8]
Normal^a^	4 (2.8) [0.8–7.0]
Abnormal^a^	140 (97) [93.0–99.2]
**Treatment**	
*No antibiotics*	71 (26) [20.5–31.1]
*Antibiotic treatment*	207 (74) [68.9–79.5]
*Penicillin V*^b^	124 (60) [52.9–66.6]
*Amoxicillin*^b^	46 (22) [16.8–28.5]
*(+/÷ Clavulanic acid)*	
*Macrolide*^b^	4 (1.9) [0.5–4.9]
*Other Antibiotic*^b^	33 (16) [11.2–21.7]

*AOM* Acute otitis media, *CI* Confidence interval

^a^% of tympanometries

^b^% of total antibiotic use

**Fig. 1:**
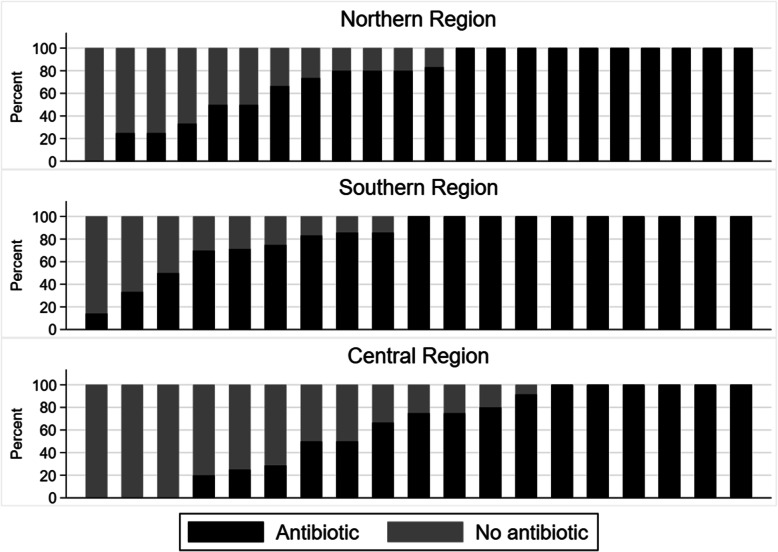
Proportion of children with AOM treated with antibiotics. - each column represents a GP practice

**Fig. 2:**
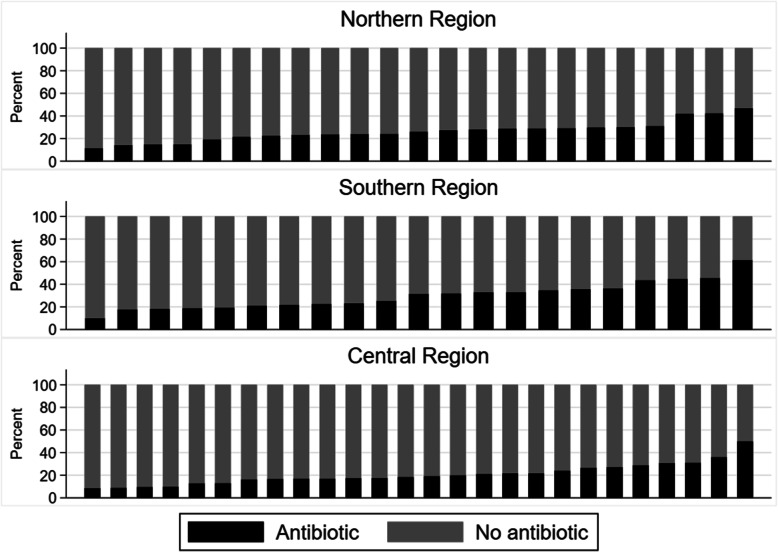
Proportion of all patients treated with antibiotics. - each column represents a GP practice

The proportion of children with AOM treated with an antibiotic varied between practices from 0 to 100% for the lowest 5th to the highest 95th percentile (Fig. 1). Also, the proportion of patients presenting with any symptoms of an acute RTI and treated with antibiotics varied from 10 to 46% among practices for the 5th and 95th percentile, respectively (Fig. 2) and among healthcare professionals between 8 and 50% (not shown).

Antibiotics were prescribed for 74% of the children with AOM. Penicillin V was used in 60% of cases. Amoxicillin was prescribed for 22% of the children. In the crude analysis of association with antibiotic treatment; fever, poor general condition and the practice’s antibiotic prescribing rate (highest vs lowest rate) to all patients with symptoms of RTI and specifically to children with AOM showed association (Table 3).

**Table 3:** Factors associated with prescription of antibiotics to children with AOM in Danish general practice

Variable	OR_**crude**_ [95% CI]	Model 1 OR_**adj**_ [95% CI]	Model 2 OR_**adj**_ [95% CI]	Model 3 OR_**adj**_ [95% CI]	Model 4 OR_**adj**_ [95% CI]
N	278	278	278	278	263
**Patient Variables**					
**Gender**					
Girls	1 (reference)	1 (reference)	1 (reference)	1 (reference)	1 (reference)
Boys	0.86 [0.50–1.49]	0.67 [0.35–1.26]	0.75 [0.38–1.46]	0.77 [0.41–1.43]	0.81 [0.41–1.60]
**Age**					
< 2 years	1 (reference)	1 (reference)	1 (reference)	1 (reference)	1 (reference)
≥ 2 years	0.63 [0.36–1.10]	0.79 [0.43–1.45]	0.85 [0.45–1.61]	0.76 [0.41–1.43]	0.82 [0.43–1.58]
**Symptoms**					
Symptom duration ≤3 days	0.63 [0.37–1.09]	0.71 [0.39–1.32]	0.70 [0.37–1.30]	0.66 [0.34–1.26]	0.74 [0.38–1.45]
Symptom worsening	0.96 [0.45–2.03]	0.67 [0.28–1.58]	0.65 [0.28–1.52]	0.66 [0.28–1.56]	0.78 [0.31–1.98]
Fever (temp. > 38.5 °)	3.47 [1.98–6.07]	3.69 [1.93–7.05]	4.01 [2.01–8.00]	3.95 [2.02–7.72]	3.72 [1.87–7.40]
Purulent ear secretion	1.84 [0.88–3.87]	2.35 [1.01–5.50]	2.39 [0.98–5.79]	2.10 [0.91–4.88]	1.96 [0.84–4.58]
Ear/face pain	0.57 [0.31–1.03]	0.79 [0.41–1.53]	0.77 [0.39–1.53]	0.71 [0.36–1.40]	0.82 [0.40–1.64]
**Clinical Findings**					
Poor general condition	4.02 [1.65–9.81]	3.12 [1.31–7.46]	2.91 [1.20–7.03]	2.87 [1.20–6.88]	2.62 [1.07–6.39]
Tympanometry performed	1.33 [0.77–2.29]	1.36 [0.76–2.42]	1.74 [0.94–3.22]	1.33 [0.74–2.37]	1.30 [0.70–2.40]
**Other**					
Parent request for antibiotics	2.12 [0.46–9.75]	1.41 [0.30–6.64]	1.43 [0.30–6.92]	1.16 [0.26–5.29]	1.33 [0.28–6.36]
**GP Variables**					
**Region**					
Northern Denmark	1.28 [0.73–2.26]		1 (reference)		
Southern Denmark	1.21 [0.68–2.15]		1.08 [0.51–2.29]		
Central Denmark	0.61 [0.34–1.09]		0.56 [0.28–1.12]		
**Gender**					
Female	1.73 [0.99–3.02]		1 (reference)		
Male	0.58 [0.33–1.01]		0.53 [0.27–1.06]		
**Age**					
< 50	0.88 [0.50–1.54]		1 (reference)		
≥ 50	1.14 [0.65–1.98]		1.84 [0.90–3.77]		
**Practice rate of antibiotic prescribing for RTIs overall**					
p (25) lowest	0.73 [0.39–1.37]			1 (reference)	
p(25)-p(75)	0.62 [0.36–1.07]			1.05 [0.50–2.20]	
p(75) highest	2.41 [1.26–4.62]			2.85 [1.07–7.60]	
**Practice rate of antibiotic prescribing for AOM**					
p (25) lowest	0.26 [0.15–0.47]				1 (reference)
p(25)-p(75)	1.91 [1.05–3.46]				2.93 [1.45–5.94]
p(75) highest	2.09 [1.07–4.08]				4.15 [1.82–9.47]
Pseudo R2	N/A	0.1254	0.1512	0.1516	0.1716
Mean VIF	N/A	1.11	1.17	1.23	1.16

*AOM* Acute Otitis Media, *OR* Odds ratio, *CI* Confidence interval, *adj* Adjusted, *GP* General practitioner, p (25): 25th percentile, p(25)-p(75): 25th to 75th percentile, p(75): 75th percentile, *VIF* Variance inflation factor

The signs and symptoms associated with antibiotic treatment were fever, purulent ear secretion, and poor general condition (Table 3, Model 1). Antibiotic prescribing was not statistically significant associated with practice location, the gender, or age of the principal GP(s) (Table 3: Model 2). Antibiotic prescribing was associated with the practice’s rate of antibiotic prescription to RTI patients in general (highest vs lowest rate OR_adj_ 2.85 (95% confidence interval (CI): 1.07–7.60)) (Table 3: Model 3) and specifically to children with AOM (middle vs lowest rate OR_adj_ 2.93 (CI: 1.45–5.94) and highest vs lowest rate OR_adj_ 4.15 (CI: 1.82–9.47)) (Table 3: Model 4).

## Discussion

### Principal findings

Three out of four children diagnosed with AOM in Danish general practice were prescribed antibiotics. The inter-practice variation was considerable. Among patient characteristics, fever, poor general condition, and purulent ear secretion were associated with antibiotic prescribing. Furthermore, the practice’s rates of prescribing antibiotics for RTIs in general and specifically for AOM in children were closely associated with prescription of antibiotics to the individual child. Parent request for antibiotics was not significantly associated with antibiotic prescribing in any of the models.

### Comparison to other studies

The clinical findings associated with the AOM diagnosis were similar to findings in other studies; purulent ear secretion, ear pain, abnormal tympanometry, and fever having the strongest associations (Supplementary Table 1) [2–4]. Only 52% of children diagnosed with AOM had a tympanometry performed, even though 55 out of 60 participating practices performed at least one tympanometry, indicative of having access to tympanometry.

We found an antibiotic prescribing rate of 74%, similar to a Danish study from year 2012 with a prescribing rate of 73% for AOM [12], an Australian study from 2017 (79%) [23], and a Swedish study from 2016 (75%) [24].

We found that penicillin V was prescribed for 60% of cases, and amoxicillin for 22%. In Denmark, the prevalence of penicillin-resistant *Streptococcus pneumoniae* is low, and since penicillin V is a narrow-spectrum antibiotic and has relatively few side effects, compared to other antibiotics, it is recommended as the first-choice antibiotic for children with AOM [25, 26]. In most other western countries, amoxicillin is the first-choice antibiotic [4, 27].

This study demonstrated large variations in Danish practices’ antibiotic prescribing rates for RTIs (Fig. 2) and specifically for AOM in children (Fig. 1). Similar variations were found in an American paediatric primary care network, indicating that unintended variation may be a general problem [28].

In this study fever, purulent ear secretion, and poor general condition were statistically significantly associated with antibiotic prescribing. Multiple studies suggest these findings to be indicative of an antibiotic prescription [2–4]. In addition, a Danish study from 2013 found type B tympanometry and a red eardrum associated with antibiotic prescribing [15]. In this study, having a tympanometry performed was not statistically significantly associated with antibiotic treatment (Table 3). The vast majority of performed tympanometries were abnormal. It is likely that most of the children without a tympanometry would have had abnormal findings if it had been performed. Performance of a tympanometry was included in the analyses as an effort to equalize the validity of the AOM diagnoses.

In our study, even though recommended indicative of antibiotic treatment neither symptom duration ≥3 days nor symptom worsening was statistically associated with antibiotic treatment [2–4]. Regarding symptom duration the statistical insignificance may be explained by underpowering of the analyses. However, most likely GPs value the present state of the child much higher than the disease history when deciding whether to prescribe antibiotics.

Though likely to influence the GP, parent’s request for antibiotics was not associated with prescribing in this study. GPs may misinterpret parent’s expectations. An Australian study from 2019 found that 86% of parents disagreed that they expected antibiotics for their child, though the GP interpreted the parent as wanting a prescription [29].

In this study, the most significant predictor of antibiotic prescribing was the practice’s antibiotic prescribing rate for RTIs and for children with AOM in particular. In line with this finding, a Danish study from 1997 showed that GPs with a high over-all prescription rate are also more likely to prescribe antibiotics [13]. Some GPs may tend to follow a set pattern were AOM is treated with antibiotics regardless of whether the criteria for prescribing are met. A reason may be poor knowledge of the guidelines for AOM. However, a conservative approach has been recommended since 1981 [30]. In this study the GPs’ gender and sex was not significantly associated though suggested to be by other studies [31], Other reasons for variation in prescribing rates may be differences in the GP’s workload [32] and in the socioeconomic status of the listed patients [33]. However, in theory none of the latter should be allowed to influence the indications for antibiotic prescribing. Lastly, some GPs may simply be unaware of the fact that they are overprescribing compared to other practices. This study does not present the final explanation for this variation.

### Strengths and limitations

The main strengths of the study are the consecutive in situ recording of all consulting children with AOM and the GP and staff’s familiarity with the recording method reducing selection and information bias [14]. A participation rate of 7.5% of all GPs in the three regions was to be expected and is in line with previous audits by the APO group.

A shortcoming is the cross-sectional study design, which impairs assessments of causality; i.e. some of the specific assessments of the children including the AOM diagnosis may in some cases have been done and certainly recorded after deciding to prescribe antibiotics and thus be influenced by the decision to treat and not the other way around.

The study has a risk of selection bias. GPs’ choice to participate in the audit may depend on workload and degree of interest in RTIs, antibiotics, and quality improvement in general. A 2009 study done in Sweden showed that GPs who chose to enrol in medical audits had significantly lower antibiotic prescription patterns for RTIs compared to their non-participating colleagues [34], indicating that the real prescribing rate in Denmark may be higher than our 74% estimate. However, the aim of the study was not to assess the absolute rate of antibiotic prescriptions, but rather to analyse factors associated with antibiotic prescribing, an aim less likely to be biased by selection. The findings of the study are applicable to other Danish GPs and likely GPs in other countries with similar health care systems. The participating GPs were comparable to the rest of the Danish GP population in the three regions (Table 1), and our main finding, the large variation in antibiotic prescribing have been shown in the UK [35] and explicitly regarding paediatric patients in the US [28].

A potential limitation is the exclusion of patients for whom it was not the first visit for the current disease. This hinders evaluation of the ‘Watchful waiting’ approach. It is however speculative whether this influences our results, considering the lack of association between antibiotic prescribing and ‘Symptom duration ≤ 3 days’ in the analyses.

The study is restricted by reporting of age in whole years instead of in months. Children below 6 months of age diagnosed with AOM should always be treated with antibiotics according to national guidelines [2]. This age group could not be specifically accounted for. Furthermore, the GPs were not requested to register otoscopy findings nor bilateralism of AOM resulting in lacking information on bulging and redness of the tympanic membrane. The lack of recording bilateralism is somewhat concerning, because this parameter is also indicative of prescribing antibiotics [2]. Bulging, redness and bilateralism might account for some use of antibiotics in the study. However, the explanatory factor might be hidden in other variables in the regression (e.g. bilateralism might result in poor general condition [36]). But is very unlikely to affect the practices’ antibiotic prescribing rates for children with AOM, and possibly could not interfere with the prescription rates for all patients due to comparably few cases of AOM.

### Implications for clinical practice and further research

Overprescribing of antibiotics for children diagnosed with AOM is evident and concerning. The inter-practice variation is large and independent of the patients’ signs, symptoms, and request for antibiotics. In order to support prudent prescribing of antibiotics for AOM, interventions should target to inform high-prescribing practices and further research should investigate more detailed practice-related factors associated with overprescribing of antibiotics to AOM.

## Conclusion

GPs’ antibiotic prescribing rates for children with AOM vary considerably even considering the of signs, symptoms, request for antibiotics, and use of tympanometry. Interventions to reduce overprescribing should be targeted high-prescribing practices.

## Supplementary information

**Additional file 1: Figure S1.** Registration template.

**Additional file 2: Figure S2.** Flowchart of the inclusion process of general practices and patients.

**Additional file 3: Table S1.** Association of acute respiratory tract infection symptoms and being diagnosed with AOM.

## Data Availability

The dataset used and/or analysed during the current study is available from the corresponding author on reasonable request.

## Abbreviations

AOM: Acute otitis media
GP: General practitioner
RTI: Respiratory tract infection
APO: Audit Project Odense
VIF: Variance inflation factor
