# Supplementary figures and images for: Prescription of antibiotics to children with acute otitis media in Danish general practice
Source: BMC Fam Pract. 2020 Aug 27;21:177. doi: 10.1186/s12875-020-01248-0 (PMC7457240; doi:10.1186/s12875-020-01248-0)

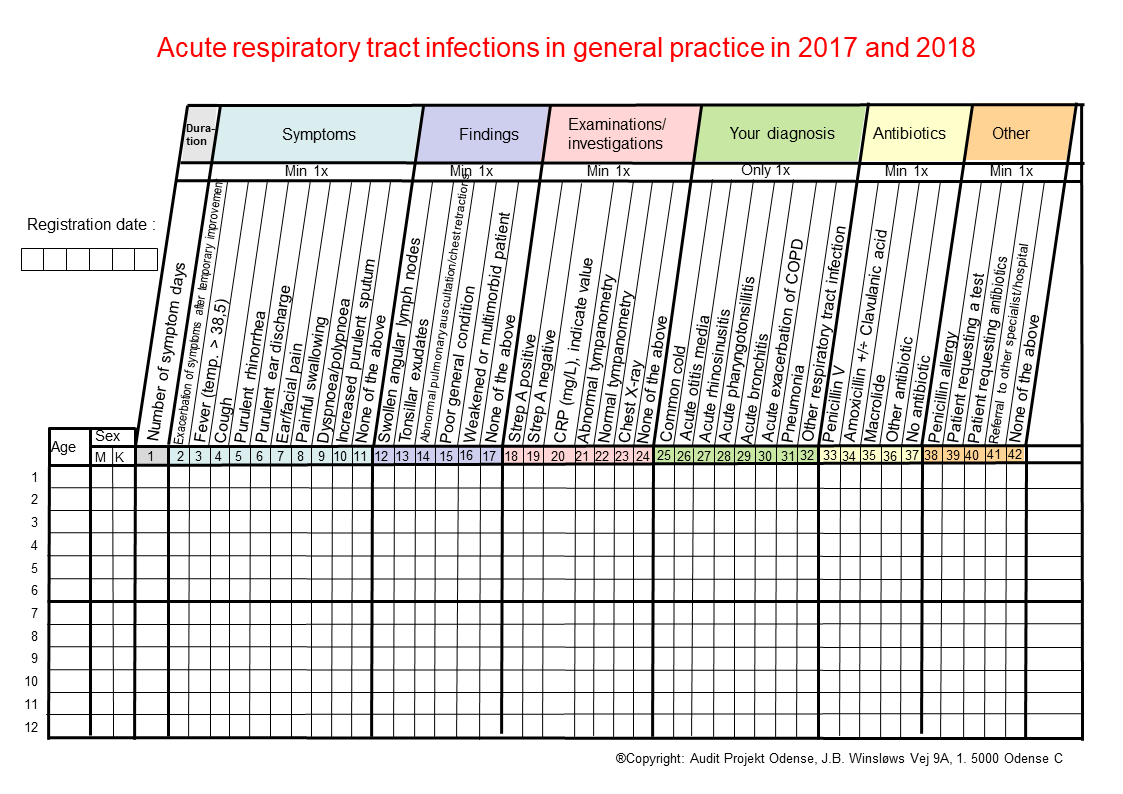

Supplement: Supplementary file 1 — Additional file 1: Figure S1. Registration template. [file 12875_2020_1248_MOESM1_ESM.tif]

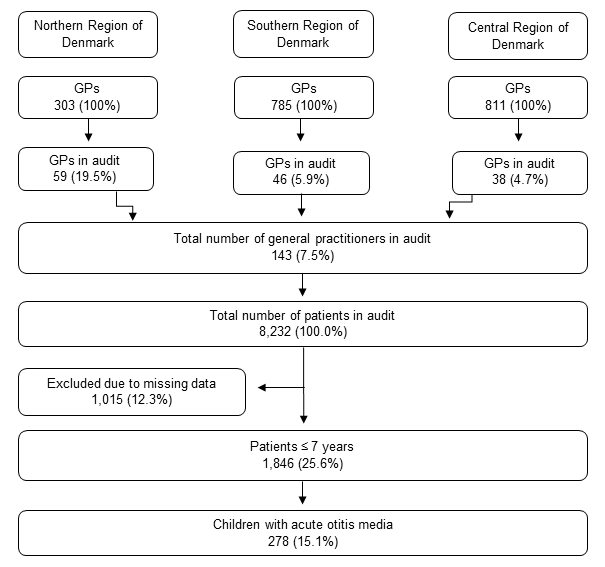

Supplement: Supplementary file 2 — Additional file 2: Figure S2. Flowchart of the inclusion process of general practices and patients. [file 12875_2020_1248_MOESM2_ESM.tif]
