# Supplementary material for: Prescription of antibiotics to children with acute otitis media in Danish general practice
Source: BMC Fam Pract. 2020 Aug 27;21:177. doi: 10.1186/s12875-020-01248-0 (PMC7457240; doi:10.1186/s12875-020-01248-0)
Supplement: Supplementary file 3 — Additional file 3: Table S1. Association of acute respiratory tract infection symptoms and being diagnosed with AOM. [file 12875_2020_1248_MOESM3_ESM.docx]

| Supplementary table 1: Association of acute respiratory tract infection symptoms and being diagnosed with AOM | | |
| --- | --- | --- |
| **Variable** | **OR_crude_ [95% CI]** | **OR_adj_ [95% CI]** |
| **Gender**  Female  Male  **Age**  ≥ 2 years  < 2 years  Symptom duration ≤ 3 days  **Symptoms**  Fever (temp. >38.5°)  Purulent ear secretion  Ear/face pain | 0.84 [0.65-1.09]  1.19 [0.92-1.54]  0.98 [0.76-1.27]  1.02 [0.79-1.32]  1.08 [0.84-1.40]  1.76 [1.35-2.28]  24.05 [13.76-42.06]  11.70 [8.80-15.57] | 1 (reference)  1.25 [0.88-1.77]  1 (reference)  1.01 [0.72-1.44]  1.02 [0.73-1.44]  2.56 [1.76-3.72]  59.40 [26.48-133.23]  11.12 [7.43-16.62] |
| **Clinical Findings**  Poor general condition | 1.79 [1.30-2.45] | 1.59 [1.00-2.52] |
| **Examinations**  Abnormal Tympanometry | 12.24 [9.07-16.52] | 5.14 [3.30-8.02] |
| Pseudo R2  Mean VIF |  | 0.3878  1.09 |
| AOM: Acute Otitis Media, adj: adjusted, OR: Odds ratio, VIF: Variance inflation factor | | |
